# Evaluation of risk factors for Suicide Attempts in Turkey’s East: A Five-Year Study

**DOI:** 10.12669/pjms.37.2.3092

**Published:** 2021

**Authors:** Turgay Bork, Abdurrahim Turkoglu, Metin Atescelik, Omer Tokgozlu

**Affiliations:** 1 Dr. Turgay Bork Department of Medicine, Firat University of Forensic Medicine, Elazig, Turkey; 2 Dr. Abdurrahim Turkoglu Department of Medicine, Firat University of Forensic Medicine, Elazig, Turkey; 3 Dr. Metin Atescelik Department of Medicine, Firat University of Emergency Medicine, Elazig, Turkey; 4 Dr. Omer Tokgozlu, Department of Medicine, Firat University of Forensic Medicine, Elazig, Turkey

**Keywords:** Forensic science, Risk factors, Suicide attempt

## Abstract

**Objectives:**

Suicide attempt has different risk factors for each community. In the study we aimed to reveal the causes of suicide attempt in Turkey’s Eastern part and to make suggestions to prevent suicide.

**Methods:**

For this study, 130 patients who were admitted to the Emergency Department of the University Hospital due to suicide attempt between January 2013 and December 2017 were included. Our University Hospital is the largest hospital in the East of Turkey. The data were obtained from hospital records and files of judicial investigations. Clinical progress records were obtained from the hospital archive. Investigation files were received from local judicial units.

**Results:**

Fifty six percent of the patients (n = 73) were female. 48% of female cases (n = 35) were married. There was major depressive disorder in 34% (n = 44) of the cases. Medicine taking was the most frequent suicide method with 63% (n = 82). The main reason for suicide was parental conflicts for female cases; and psychiatric and financial problems for males.

**Conclusion:**

Family therapy for married individuals would reduce suicide attempts in females. Psychiatric history is an important risk factor and it should be ensured that these patients are followed up regularly by the health institutions and their relatives. To prevent drug abuse, there should not be too much medicine in homes. If medicine is available at home, it should be kept in a safer environment.

## INTRODUCTION

Suicides and suicide attempts are important public health problems worldwide.^1^ According to the report of the World Health Organization, 804,000 people died of suicide in 2012, and the suicide rate was reported to be 44.2 per 100,000 people per year.^2^ As of 2016, the suicide rate in Turkey was determined to be 3.86 per 100,000 people.^3^ When we look at suicide rates around the world, there are significant differences between countries. Religious, cultural and social differences between countries create this situation.^4^ Studies reveal that there are different risk factors among societies. In some studies, unemployment and urban life,^5^ female gender and low education level^6^ are indicated as risk factors. Therefore, each society should establish its own risk factors and develop its own strategies in order to prevent suicide attempts.^7^ In the study we aimed to reveal the causes of suicide attempt in Turkey’s Eastern part and to make suggestions to prevent suicide attempt.

## METHODS

This study included suicide attempt cases admitted to the Emergency Department of Firat University Hospital between January 2013 and December 2017. Firat University Hospital is the reference hospital providing services to four cities and 1,344,067 people in the Eastern Anatolia region of Turkey. Because it is a university hospital, an advanced registration system is implemented. Patient data were obtained from records, forensic investigation files and by contacting the patients when necessary. All cases admitted for suicide attempt were included in the study. There were 130 cases in five years. Demographic data (age, gender, marital status, educational status, place of residence and psychiatric history), clinical status, admitted service, suicide cause and method were investigated. Data were categorized according to gender to determine the risk factors for both sexes.

The obtained data were transferred to Statistical Package for Social Science (SPSS) 17.0 for Windows. The descriptive statistics of the data were tabulated as mean (average) ± standard deviation, number and percentage.

### Ethical approval

The study was approved by the Firat University Ethics Committee (decision dated 05/16/2016; ethics committee number, 15–81/2).

## RESULTS

In total, 130 patients were admitted to the emergency department due to suicide attempts. Of them, 10 (7.6%) died in the emergency room during treatment. The mean age was 32.24 ± 14.12 years, and 56.2% (n = 73) of the patients were females. Further, 60.0% (n = 78) of the patients were in the age group of 21–40 years. Additionally, 47.9% of the women (n = 35) were married, and 49.1% (n = 28) of the men were single. Moreover, 73.1% of the patients (n = 95) were living in the city center. Of the total patients, 33.8% (n = 44) had a history of major depressive disorder.

Drug intoxication was found to be the most common suicide method with 63.4% (n = 82), and 80.8% (n = 59) of the women and 40.4% (n = 23) of the men were brought to the emergency department due to taking medication. The most common cause of suicide was marital conflict in women (41.1%, n = 30) and psychiatric disorder (29.8%, n = 17) and financial problem (21.1%, n = 12) in men.

## DISCUSSION

Although the concept of suicide is as old as the history of mankind, it continues to be an unresolved problem.^8^ Many countries make evaluations based on their own cases and recommend solutions. An important factor reported in suicide attempts is gender difference.^9^ For this reason, we present our data by comparing male and female gender.

Studies have shown that women are at a greater risk for suicide attempts.^10^ 10 However, men die more often due to suicides than women.^11^ This situation is reported to be due to the selected suicide methods and the fact that men are more determined to end their lives.^12^,^13^ Our data were consistent with literature. Of the total patients, 56.2% (n = 73) were female. Additionally, 7.6% (n = 10) of the patients died during treatment. Of these, 7 were male and three were female.It was found that 60% (n = 78) of the patients were in the age group of 21−40 years. In studies, adolescents and young adults are reported as high-risk groups.^14^

In various studies, being single appears to be a risk factor for both genders.^15^ Contrary to these findings, we found that most of the women were married, and most of the men were single among our patients. This was due to the reasons why women commit suicide because the women involved in this study most frequently attempted suicide due to marital conflict.

Depression is the most common mental disorder reported in individuals who attempt suicide.^16^ In the study by Suominen et al. on individuals who attempted suicide, the prevalence of major depression was reported to be 67%.^17^ In this study, 33.8% (n = 44) of the patients had a history of major depression. Major depression appears to be an important risk factor for suicide attempts in all countries.

Drug intoxication is the most preferred method of suicide.^18^ In this study, 63.4% (n = 82) of the patients were admitted to emergency department due to drug intoxication. Taking medication is a method that is more easily applied than other suicide methods and results in lower mortality. In studies, it appears to be the method of suicide chosen by women in particular.^19^ In this study, 80.8% of women and 40.4% of men were admitted to the hospital due to drug intoxication.

**Table-I:** Demographic variables and factors associated with suicide attempts.

*Variable*	*Female (N=73) Number (%56.2)*	*Male (N=57) Number (%43.8)*	*Total (N=130) Number (%100.0)*
***Age Group (year)***			
<20	11 (%15.1)	10 (%17.6)	21 (%16.2)
21-40	52 (%71.2)	26 (%45.6)	78 (%60.0)
41-60	10 (%13.7)	13 (%22.8)	23 (%17.8)
>60	-	8 (%14.0)	8 (%6.2)
***Marital Status***			
Single	31 (%42.5)	28 (%49.1)	59 (%45.4)
Married	35 (%47.9)	15 (%26.4)	50 (%38.5)
Divorced	3 (%4.1)	8 (%14.0)	11 (%8.5)
Unknown	4 (%5.5)	6 (%10.5)	10 (%7.6)
***Educational Status***			
Unable to read and write	2 (%2.7)	1 (%1.8)	3 (%2.3)
Read and write	1 (%1.4)	3 (%5.3)	4 (%3.1)
Elementary school	16 (%21.9)	13 (%22.8)	29 (%22.3)
Middle school	7 (%9.6)	6 (%10.5)	13 (%10.0)
High school	14 (%19.2)	17 (%29.8)	31 (%23.8)
University	9 (%12.3)	3 (%5.3)	12 (%9.3)
Unknown	24 (%32.9)	14 (%24.6)	38 (%29.2)
***Region of District***			
Urban	55 (%75.3)	40 (%70.2)	95 (%73.1)
Rural	18 (%24.7)	17 (%29.8)	35 (%26.9)
***Clinical Psychiatric Data***			
Major depressive disorder	23 (%31.5)	21 (%36.8)	44 (%33.8)
Bipolar disorder	-	2 (% 2.7)	2 (%1.5)
Schizophrenia	1 (% 1.4)	5 (% 8.8)	6 (%4.5)
History of suicide attempt	1 (% 1.4)	-	1 (%0.8)
Anxiety disorder	17 (% 23.3)	7 (%12.3)	24 (%18.6)
Abuse of narcotic	3 (%4.1)	6 (%10.5)	9 (%7.0)
Unknown	26 (%35.6)	18 (%31.6)	44 (%33.8)

***Variable***	***Female (N=73) Number (%56.2)***	***Male (N=57) Number (%43.8)***	***Total (N=130 ) Number (%100.0)***

***Methods of suicidal behavior***			
Hanging	3 (%4.1)	9 (%15.8)	12 (%9.0)
Drug overdose	59 (%80.8)	23 (%40.4)	82 (%63.4)
umping from high place	4 (%5.5)	5 (%8.8)	9 (%6.9)
Firearm	-	3 (%5.3)	3 (%2.3)
Cutting/stabbing object	5 (%6.8)	13 (%22.8)	18 (%13.9)
Corrosive substance drinking	2 (%2.7)	2 (%3.5)	4 (%3.0)
Narcotic poisoning	-	2 (%3.5)	2 (%1.5)
***Reason for suicide***			
Family problems	30 (%41.1)	8 (%14.0)	38 (%29.2)
Financial problems	5 (%6.8)	12 (%21.1)	17 (%13.1)
Psychiatric diseases	22 (%30.1)	17 (%29.8)	39 (%30.0)
Love problems	2 (%2.7)	7 (%12.3)	9 (%6.9)
Unknown	14 (%19.2)	13 (%22.3)	27 (%20.8)
***Clinical Status***			
Hospitalization	49 (%67.1)	37 (%64.9)	86 (%66.2)
Discharged from Emergency	21 (%28.8)	13 (%22.8)	34 (%26.2)
Death	3 (%4.1)	7 (%12.3)	10 (%7.6)
***Clinic***			
No service	24 (%32.9)	20 (%35.1)	44 (%33.9)
Plastic surgery	1 (%1.4)	4 (%7.0)	5 (%3.8)
Pyschiatry	1 (%1.4)	1 (%1.8)	2 (%1.5)
Brain surgery	2 (%2.7)	0	2 (%1.5)
Pediatri	2 (%2.7)	0	2 (%1.5)
Orthopedy	1 (%1.4)	2 (%3.5)	3 (%2.3)
General surgery	0	2 (%3.5)	2 (%1.5)
Intensive care	27 (%37.0)	20 (%35.1)	47 (%36.2)
Emergency service	6 (%8.2)	1 (%1.8)	7 (%5.4)
Leave the emergency	9 (%12.3)	7 (%12.2)	16 (12.4)

Studies have shown that the causes of suicide also vary in different societies. In the study of McCaig et al.^20^, substance abuse was reported as the most common cause of suicide. Ayehu et al.^15^ reported family problems as the most common cause of suicide. In this study, the most common cause of suicide was family problems in women (41.1%, n = 30) and psychiatric disorders (29.8%, n = 17) and financial problems (21.1%, n = 12) in men. These differences can occur because the expectations and characteristics of individuals in each society are different. For this reason, suicide prevention measures differ between societies.

### Limitations of the study

The requested information could not be obtained in all cases. For example, educational status, the reason for suicide. Communication was attempted with these. However, some of them could not be contacted due to the address or phone change. These are highlighted.

## CONCLUSION

Marriage and presence of marital conflict is a risk factor for suicide attempts in women. Therefore, providing counselling to all married individuals will be a protective measure against these suicides. Psychiatric history appears to be an important risk factor for suicide attempts for all individuals. Therefore, healthcare institutions should monitor patients closely and regularly, and their relatives should be warned regarding this problem. Prone individuals should not keep medications at home, or the drugs should be kept in more secure places in houses where there are possible patients.

### Authors’ Contribution:

**TB:** Conceptualized and designed the study, revised the manuscript and approved the final version. He is responsible for accuracy and integrity of work.

**AT & OT:** Did the data entry and statistical analysis.

**MA:** Did data collection.
